# Ion-Induced Dipole Interactions Matter in Metadynamics Simulation of Transition Metal Ion Transporters

**DOI:** 10.1021/acs.jctc.4c01535

**Published:** 2025-04-03

**Authors:** Majid Jafari, Luca Sagresti, Jian Hu, Kenneth M. Merz

**Affiliations:** † Department of Biochemistry & Molecular Biology, Michigan State University, East Lansing, Michigan 48824, United States; ‡ Scuola Normale Superiore, Piazza dei Cavalieri 7, I-56126 Pisa, Italy and CSGI; § Istituto Nazionale di Fisica Nucleare (INFN) sezione di Pisa, Largo Bruno Pontecorvo 3, 56127 Pisa, Italy; ∥ Department of Chemistry, Michigan State University, East Lansing, Michigan 48824, United States

## Abstract

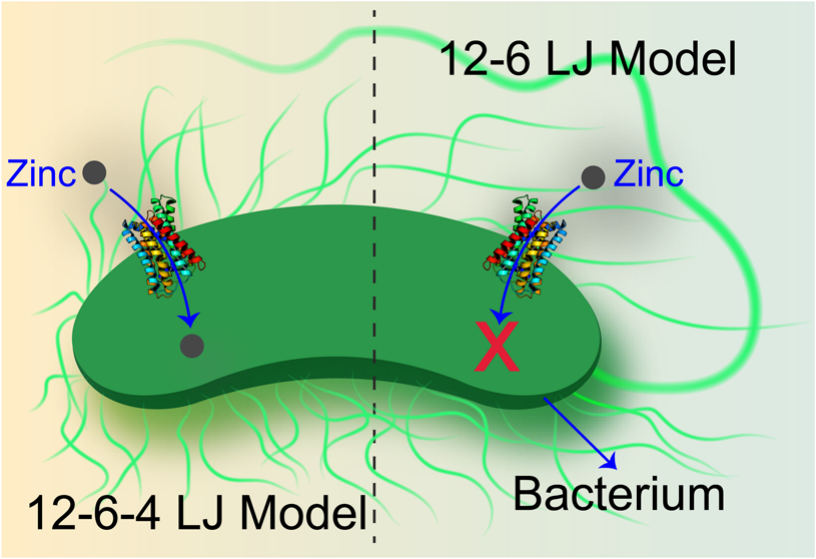

Metal transporters play crucial roles in the homeostasis and detoxification of beneficial and toxic metals in the human body. Due to experimental limitations in studying some metal transporters, numerous simulation studies have been conducted to understand the mechanisms of metal transport. However, studying the transport of divalent metal ions across the plasma membrane by metal transporters has been challenging with traditional molecular dynamics (MD) simulations. The metal ions often become trapped inside the transporter due to encountering high energy barriers during the transport process. In this study, we combined a recently developed metadynamics setup, known as well-tempered (WT) volume-based MTD, with the 12-6-4 Lennard-Jones (LJ) model representing transition metal-His/Asp/Glu side chain interactions. We used this approach to investigate the mechanism of action of a Zrt-/Irt-like protein (ZIP) transporter and compared the results with simulations using standard 12-6 LJ parameters for the transition metal-His/Asp/Glu side chain interactions. Our results show that the 12-6-4 LJ model for transition metal-His/Asp/Glu side chain interactions samples conformational space more broadly than the standard 12-6 LJ model for the same interactions in MTD simulations, facilitating the sampling of states that are hard to reach with the standard 12-6 model within the same time scale. This is even more remarkable given the fact that the model is dominated by 12-6 LJ interactions for the majority of the system, while the transition metal-His/Asp/Glu side chain interactions are the only interactions using the 12-6-4 LJ model. Hence, a small subset of interactions significantly modifies the states sampled by the entire protein leading to a more frequent observation of the transport of the transition metal ion. Overall, using 12-6-4 LJ to model the transition metal-His/Asp/Glu side chain interactions increases the potential for discovering additional metastable states by enabling metal ions to traverse more freely along the defined transport pathways.

## Introduction

Metadynamics (MTD) is an enhanced sampling method wherein a history-dependent potential is added to the system as a function of collective variables (CVs) to facilitate the exploration of rare events.^1−4^ This method reconstructs a free energy surface (FES) for the system using reweighting algorithms based on the biasing potential, which is the negative image of the applied bias potential.^2,4^ One of the primary challenges of classical molecular dynamics (MD) simulations is the tendency of the system to become trapped in local energy minima because of the high free energy barriers.^2,3^ The time required to overcome energy barriers and visit rare events cannot be effectively addressed in most complex systems using regular MD simulations, given current computational resources. In other words, regular MD simulations are insufficient for complex processes, which usually occur on the order of a few milliseconds to seconds.^2,5^ Therefore, it is necessary to drive the system toward rare events using external biasing potentials, as done in MTD simulations.

Since its introduction, numerous MTD variants have been developed, including well-tempered (WT)-MTD,^6,7^ parallel tempering (PT)-MTD,^8^ and stochastic resetting (SR)-MTD methods.^9^ Capelli et al. introduced a new set of CVs to be used alongside WT-MTD to estimate ligand binding affinity even when multiple escape pathways exist.^10^ This new procedure was tested on the lysozyme T4 enzyme containing a ligand (benzene) and successfully estimated the binding free energy of the ligand, in agreement with the reported experimental data. The technique successfully rediscovered all previously determined ligand binding pathways and uncovered three new binding pathways that had not been identified before.^10^

However, if one wishes to use enhanced sampling techniques to study metalloproteins with standard 12-6 Lennard-Jones (LJ) parameters representing transition metal-His/Asp/Glu side chain interactions, the results may not be accurate.^11−13^ For example, the 12-6 LJ model either underestimates or overestimates the metal ion interactions with water molecules and coordinating residues.^11−13^

Although the standard 12-6 LJ nonbonded model offers a quick approach for simulating ion-containing systems with traditional force fields, there are limitations that should be considered when applying this model.^14^ This model is unable to simultaneously reproduce experimental hydration free energies (HFE) and ion-oxygen distances (IOD) values for metal ions.^11^ Furthermore, in complex systems, particularly those with high ion concentrations, inaccurate estimations of VDW interactions may occur.^14^ To address this issue, researchers often modify the combining rules to approximate LJ potential parameters or adjust the parameters to improve the reliability of the simulation results.

Li and Merz developed a new model known as the 12-6-4 LJ model in 2013.^11^ The new 12-6-4 LJ nonbonded model^11^ extends the standard 12-6 LJ nonbonded model.^14−17^ This new model introduces an r^–4^ term to the standard 12-6 LJ model to account for ion-induced dipole interactions between metal ions and water molecules. It specifically addresses the dipole-induced dipole and charge-induced dipole interactions, which are not explicitly accounted for in the standard 12-6 model. Moreover, the new model accurately reproduces experimental values, including HFE, IOD, and coordination numbers (CN) for various divalent metal ions.^11^ In general, the model achieves a balance between computational efficiency and accuracy, making it a valuable model for biomolecular research.

Since its development, the 12-6-4 LJ model has been used to develop finely tuned parameters for molecules other than water-metal ions, such as imidazole-metal ion,^12^ acetate-metal ion,^13^ and phosphate-metal ion^18^ complexes. These parameters accurately reproduce experimental free energy interactions between the specified molecules and metal ions, while the standard 12-6 LJ model either underestimates or overestimates the strength of these interactions. Therefore, these parameters refine the modeling of interactions between histidine, negatively charged residues, and metal ions in complex biomolecular systems like metalloproteins and metal ion transporters.^12,13,18^ The improved parameters result in greater accuracy when compared to experimental results, paving the way for more accurate computation of the underlying FES in even more complex systems. Thus, a reliable FES can be extracted either through brute force MD simulations or by using techniques that accelerates the sampling.

The Zrt-/Irt-like protein (ZIP) family is a class of divalent metal transporters crucial for the uptake of essential trace elements.^19−22^ In addition to their key roles in maintaining the homeostasis of beneficial trace minerals, such as zinc, manganese, and iron, some ZIPs are also involved in the absorption of toxic metals, including cadmium.^23−25^ Nevertheless, zinc ion is believed to be the primary substrate for most ZIPs. Dysregulation of ZIP transporters leads to disrupted metal homeostasis and has been linked to many diseases.^20,21,26^

**Figure 1:**
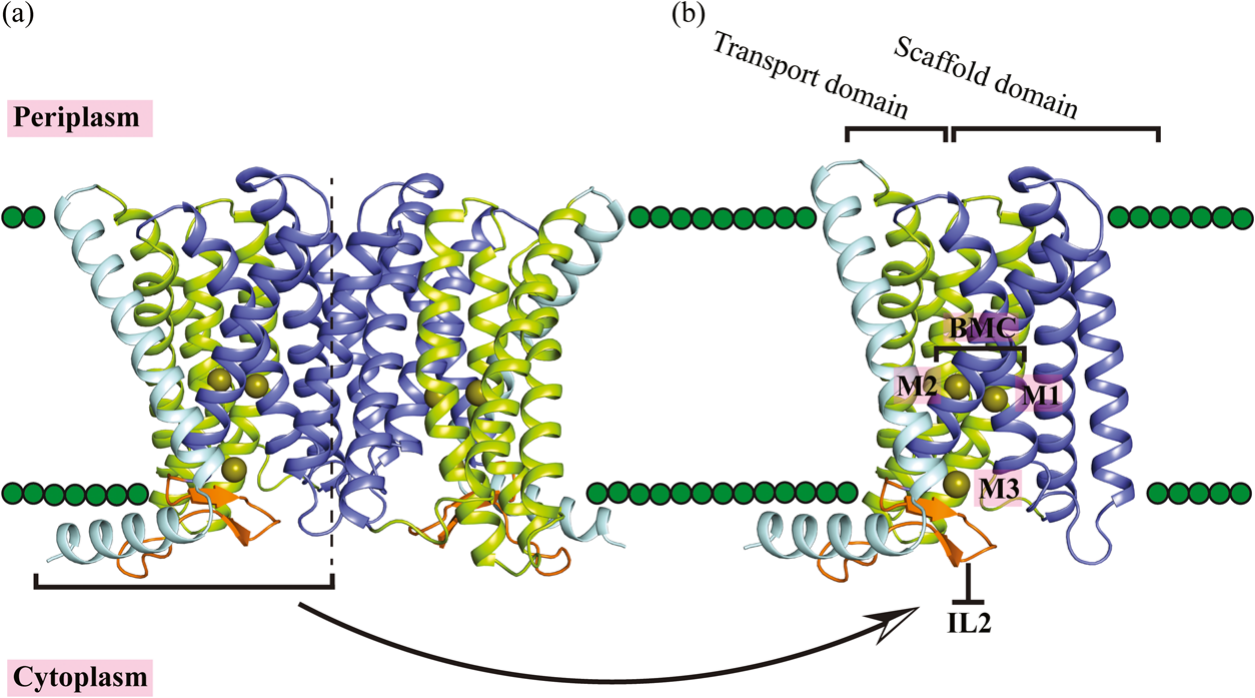
Cryo-EM structure of the zinc transporter BbZIP used in the MTD simulations with its metal binding sites. (a) The dimer structure of BbZIP (PDB ID 8GHT). (b) A monomeric BbZIP with highlighted elements, including the transport domain (green), the scaffold domain (purple), and the IL2 loop (orange). The zinc binding sites M1, M2, and M3 are indicated. The N-terminal amphipathic helix and the adjacent TM0 are typically not considered part of the transport domain, so they are colored pale cyan. The green spheres represent the phospholipid headgroups of the lipid bilayer.

Evolutionary studies have shown that eukaryotic ZIPs evolved from prokaryotic ancestors, and their structural features and transport mechanisms are believed to be largely conserved.^19,27^ The crystal structure^28^ of a ZIP from *Bordetella bronchiseptica* (BbZIP) revealed a novel transporter fold, which consists of eight transmembrane helices (TM) with two TMs involved in metal binding (TM4 and TM5) being sandwiched by TM1–3 and TM6–8. The structure also revealed a binuclear metal center (BMC, including the M1 and M2 sites) with two metal ions coordinated by the conserved metal-chelating residues from TM4 and TM5 in the middle of the transport pathway. The cryo-EM structure of a BbZIP dimer was later resolved,^29^ revealing a third ion at an egress site (the M3 site) facing the cytoplasm, which is coordinated by two carboxylic acid residues (D144 and E276 from TM3 and TM7, respectively) and two histidine residues (H149 and H151) in the second intracellular loop (IL2)^29^ (Figure 1). It has been shown that M1 is essential for metal transport whereas M2 is dispensable. M2 deletion does not affect protein structure but moderately reduced transport activity.^30^ The M3 site has been proposed to function as a zinc sensor and metal sink to regulate the release of the metal substrate into the cytoplasm.^29,31^

BbZIP has been proposed to transport zinc through an elevator-type transport mechanism.^32,33^ In this mode, the scaffold domain (TM2/3/7/8) serves as a static structure, while the transport domain (TM1/4/5/6) moves upward and downward relative to the scaffold. When the transport domain is in the outward-facing conformation, the transport site is open to the periplasm, allowing zinc ions to bind to the BMC. Then, the transport domain moves downward and enables the transporter to adopt the inward-facing conformation. After this transition, the zinc ions are released to the cytoplasm via a metal relay.^28,29,31,32^

Here, we studied the mechanism of zinc transport in BbZIP (PDB ID 8GHT) (Figure 1) using standard 12-6 LJ parameters for transition metal-His/Asp/Glu side chain interactions and compared them to the corresponding 12-6-4 LJ parameters^11−13^ combined with volume-based WT-MTD.^10^ The aim of the current study was to investigate the impact of the finely tuned parameters of the 12-6-4 LJ model on MTD simulations. We integrated the 12-6-4 LJ model with the WT volume-based MTD method and the results demonstrated that the finely tuned parameters for the transition metal-His/Asp/Glu side chain interactions increase the likelihood of observing rare events in BbZIP. The application of these parameters allowed us to observe the elevator motion of the metal transporter, a rare event, more frequently, with respect to standard the 12-6 LJ model for transition metal-His/Asp/Glu side chain interactions, given the same amount of simulation time.

## Methods

### Note on Terminology

Here we will use the term “12-6 LJ model” to generically mean the 12-6 LJ parameter set used to model transition metal-His/Asp/Glu side chain interactions and we will use the “12-6-4 LJ model” to generically mean the 12-6-4 LJ parameter set used to model transition metal-His/Asp/Glu side chain interactions. Indeed, this work is specifically about comparing the 12-6 LJ and 12-6-4 LJ parameters for transition metal-His/Asp/Glu side chain interactions and how this parameter choice makes a difference in MTD simulations of the ZIP transporter.

### The 12-6-4 LJ Nonbonded Model

The nonbonded interaction potential between two particles in the AMBER force field is defined by the following equation.^11,14,34,35^
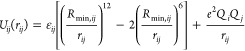
where the well depth of the LJ potential, the equilibrium distance between particles *i* and *j*, and the distance between particles *i* and *j* are represented as ε_*ij*_, *R*_min*,ij*_, and *r*_*ij*_, respectively. *Q*_*i*_ and *Q*_*j*_ represent the partial charges of particles i and j, respectively, and *e* indicates the proton charge.

Although polarizable force fields provide a more realistic description of charge transfer, induced dipole, and dipole interactions, they are more computationally demanding than traditional force fields, which simplify the description of these interactions.^36,37^ This efficiency of classical force fields is most apparent in large simulation systems especially for a system as large as the one used in the current work. The interaction between metal ions and surrounding molecules can be further complicated by charge transfer and polarizability effects, which cannot be easily handled by classical force fields. To retain computational efficiency while increasing the accuracy of the standard 12-6 LJ model in classical force fields, we used the 12-6-4 LJ model to account for the interactions of divalent metal ions with their surroundings.^11−13^ This approach allows for a more accurate system representation with minimal computational cost.

In the 12-6-4 LJ model,^11^ the following equation is used to describe nonbonded interactions, with the last part in the brackets representing the C_4_ term
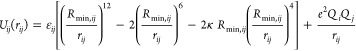
This model uses the following equation to estimate the C_4_ terms between metal ions and water molecules
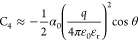
where θ is the angle formed by the metal ion, the induced dipole, and the metal ion’s electronic field. α_0_, *q*, and ε represent the polarizability of the water molecule, the charge of the metal ion, and the dielectric constant, respectively.

The C_4_ parameters recommended in a previous study^11^ were used to handle the interactions between water molecules and the metal ion. The C_4_ parameters for the imidazole nitrogen–metal ion and acetate oxygen–metal ion were also obtained from previous studies.^12,13^

### The MD Simulation Setup and Configuration

The coordinates of BbZIP were obtained from the PDB RCSB database (PDB ID 8GHT).^29^ The cadmium metal ions were replaced with zinc ions. To ensure that this replacement did not significantly alter the cryo-EM structure, the system underwent minimization and equilibration phases. Then, it was compared to cryo-EM structure containing cadmium ions. The transporter dimer was then placed in a lipid bilayer composed of 200 lipid molecules. Each membrane leaflet consisted of 67 POPE and 33 POPG molecules to mimic the lipid bilayer of Gram-negative bacteria. TIP3P water molecules^38^ were added to the systems, covering 25 Å above and below the membrane-protein systems. Additionally, 150 mM KCl was added to neutralize the systems. Each system underwent a five-stage minimization phase, followed by 5 ns of NVT and 5 ns of NPT ensembles. The equilibrated simulation systems were then used to run the WT volume-based MTD simulations for 500 ns using the ff19SB^39^ and lipid21^40^ force fields, along with the Joung and Cheatham parameters for counterions.^41^ The Berendsen barostat^42^ with semi-isotropic pressure coupling at 1 bar and a pressure relaxation time of 1.0 ps was applied to maintain the pressure. The Langevin dynamics algorithms with a friction coefficient of 1.0 ps^–1^ was used to maintain the temperature at 303 K. A time step of 0.002 ps was used, and the SHAKE algorithm^43^ was employed to constrain covalent and hydrogen bonds. The simulations were conducted using periodic boundary conditions with a nonbonded cutoff of 9.0 Å. The Particle Mesh Ewald (PME) was used for handling long-range electrostatics. The simulation data was recorded every 10,000 steps for restart files, every 2500 steps for coordinates, and every 250 steps for energies. The AMBER simulation package, version 22,^44^ together with the community developed software PLUMED v.2.8,^45,46^ were used to perform the simulations. The simulation systems were generated using the CHARMM-GUI server.^47^

In light of the significant role of the IL2 loop in regulating metal transport and release, four simulation setups were designed to explore all potential pathways the metal ion could take within the transporter during release. In the first setup, the metal ion occupied the M1 and M2 binding sites while the loop was unfolded, and the M3 binding site was empty. In the second setup, all metal binding sites were occupied, and the loop was folded. In the third setup, the loop was folded with the metal ion occupying the M1 and M2 sites and the M3 site empty. In the final setup, the M3 and M1 were occupied by the metal ion, while the M2 site remained empty. To prepare the system for the first setup, we unfolded the loop using steered molecular dynamics (SMD). The system underwent energy minimization and equilibration following the procedure described above. Afterward, the loop was gradually pulled toward the cytoplasm employing a force constant of 1000 kcal/(mol•Å^2^) over a 100 ns SMD simulation period.

We used the WT volume-based MTD method to sample release pathways of the metal ion within the transporter. This method efficiently identifies metal ion release pathways without prior knowledge of these pathways or the bound state of the metal ion. The starting point is to define a particular spherical volume that enclose the transporter, taking into account especially the main pathway followed by the metal movement suggested in previous experimental studies. Then, a repulsive potential at the boundary of this spherical volume was applied to limit the sampling space. The CVs were the three spherical coordinates (ρ, θ, φ), where ρ represents the radial distance of one of the metal ions in the transporter (Zn^2+^) from the center of mass of the transporter, depending on the setup. θ is the polar angle, and φ is the azimuthal angle. The bias is applied within a sphere of finite radius centered around the transporter’s center of mass using a harmonic restraint of 100 kcal/mol Å^2^, defining the volume in which the metal ion binding and unbinding pathways are explored. Furthermore, the metal ion coordination number was used as a CV to measure whether the metal ion is in contact with the heavy atoms of the transporter. The coordination number is determined by the following switching function that considers the distance between the non-hydrogen atoms of the transporter and the metal ion, with a threshold distance defining a formed contact
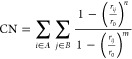
where the contact is defined using a cutoff of *r*_0_ = 4 Å, with the exponentials set to *n* = 8 and *m* = 16.

The following parameters were set to perform WT-MTD simulations bias factor = 10, hill height = 1 kcal/mol, σ_rho_ = 0.5 Å, σ_theta_ = π/16, σ_phi_ = π/8, and deposition time = 5 ps.

The FES was reconstructed using reweighting algorithms with the metal ion distance from the spherical volume center of mass (CV1(ρ)) and the metal ion coordination number (CV2).

## Results and Discussion

### Elevator Mechanism Observed in Metadynamics Simulations

In general, the BbZIP dimer consists of several key components. The scaffold domain, consisting of TM2, TM3, TM7, and TM8 and mediating dimerization, remains relatively static during the metal transport cycle (Figure 1). The transport domain (elevator motion domain), composed of TM1 and TM4–6, changes its conformation between the outward-facing and inward-facing states while transporting the metal ion. The BMC (M1 and M2) and M3 sites which are the metal binding sites. Finally, an intracellular histidine-rich loop (IL2) located between TM3 and TM4 that folds back into the inward-facing pocket and functions as a zinc sensor, blocking zinc import when intracellular zinc concentrations are high.^31^

In all simulation setups (Table 1), a bias potential was applied to the metal ion at the M1 binding site to displace it.

**Table 1:** System Setup Information and Summary of MTD Simulation Results, Including the Metal Ion Position after a 500 ns MTD Simulation for Each Simulation Replicas

**conditions**	**simulation replicas**	**metal release or retention using** 12-6-4 **LJ**	**metal release or retention using** 12-6 **LJ**
**M1 and M2 loaded with Zn2+; M3 empty; IL2 unfolded (first setup)**	1	cytoplasm	moves toward M2 and stays above it
	2	cytoplasm	moves toward M2 and stays above it
	3	cytoplasm	moves toward M2 and stays above it
	4	N/A^a^	toward cytoplasm, not released
	5	N/A	cytoplasm through pathway 2
	6	N/A	cytoplasm through pathway 1
**M1, M2, and M3 loaded with Zn**^**2+**^**; IL2 folded (second setup)**	1	periplasm	remains around M1
	2	cytoplasm	remains around M1
	3	cytoplasm	moves toward M2 and stays above it
	4	cytoplasm	cytoplasm through pathway 2
	5	cytoplasm	periplasm
	6	cytoplasm	remains around M1
**M1 and M2 loaded with Zn2+; M3 empty; IL2 folded (third setup)**	1	cytoplasm	remains around M1
	2	periplasm	remains around M1
	3	toward cytoplasm, then moves back to M1 and remains there	remains around M1
	4	N/A	toward cytoplasm, not released
	5	N/A	remains around M1
	6	N/A	remains around M1
**M1 and M3 loaded with Zn**^**2+**^**; M2 empty; IL2 folded (fourth setup)**	1	periplasm	remains around M1
	2	cytoplasm	moves to and remains at M2
	3	remains around M1	remains around M1
	4	periplasm	remains around M1
	5	N/A	remains around M1
	6	N/A	toward cytoplasm, not released

a N/A indicates that no simulation was performed in that replica.

The metal ion could then move either toward the cytoplasm or periplasm. The protein structure used in this study was a homodimer of BbZIP in an inward-facing conformation, indicating the transport domain was oriented inward.

**Figure 2:**
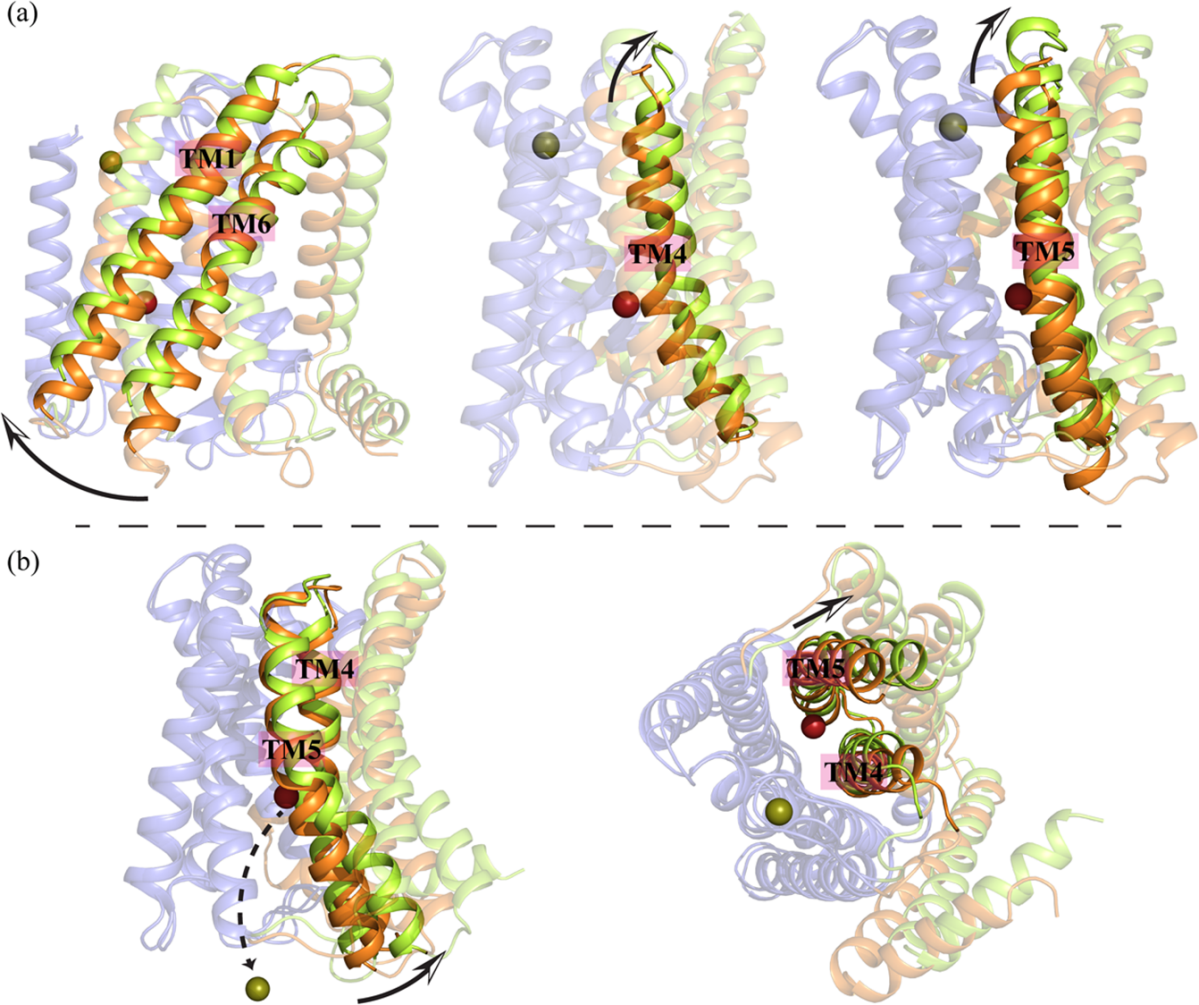
Transporter elevator motion and metal release into the cytosol observed in MTD simulations using 12-6 LJ parameters. (a) Depicts the mechanism of zinc transport in the reverse direction and the structural rearrangement of the transporter during this process in the second simulation setup (See Table 1) using 12-6 LJ parameters. The metal is released into the periplasm as TM1 and TM6 swing to the right, while the periplasm face of TM4 and TM5 move away from the main tunnel. (b) Shows the release of the metal ion into the cytoplasm, with the cytoplasmic portion of TM5 moving away from the main tunnel.

Out of 24 MTD simulations performed using standard 12-6 LJ parameters, only one replica (the fifth replica of the second setup) exhibited the elevator motion mechanism (Table1). In this replica, we observed the TM1 and TM6 helices swinging leftward and moving upward toward the periplasm, while TM4 and TM5 moved both upward and away from the periplasmic part of the main tunnel and upward to provide space for the metal to be released (Figure 2a).

**Figure 3:**
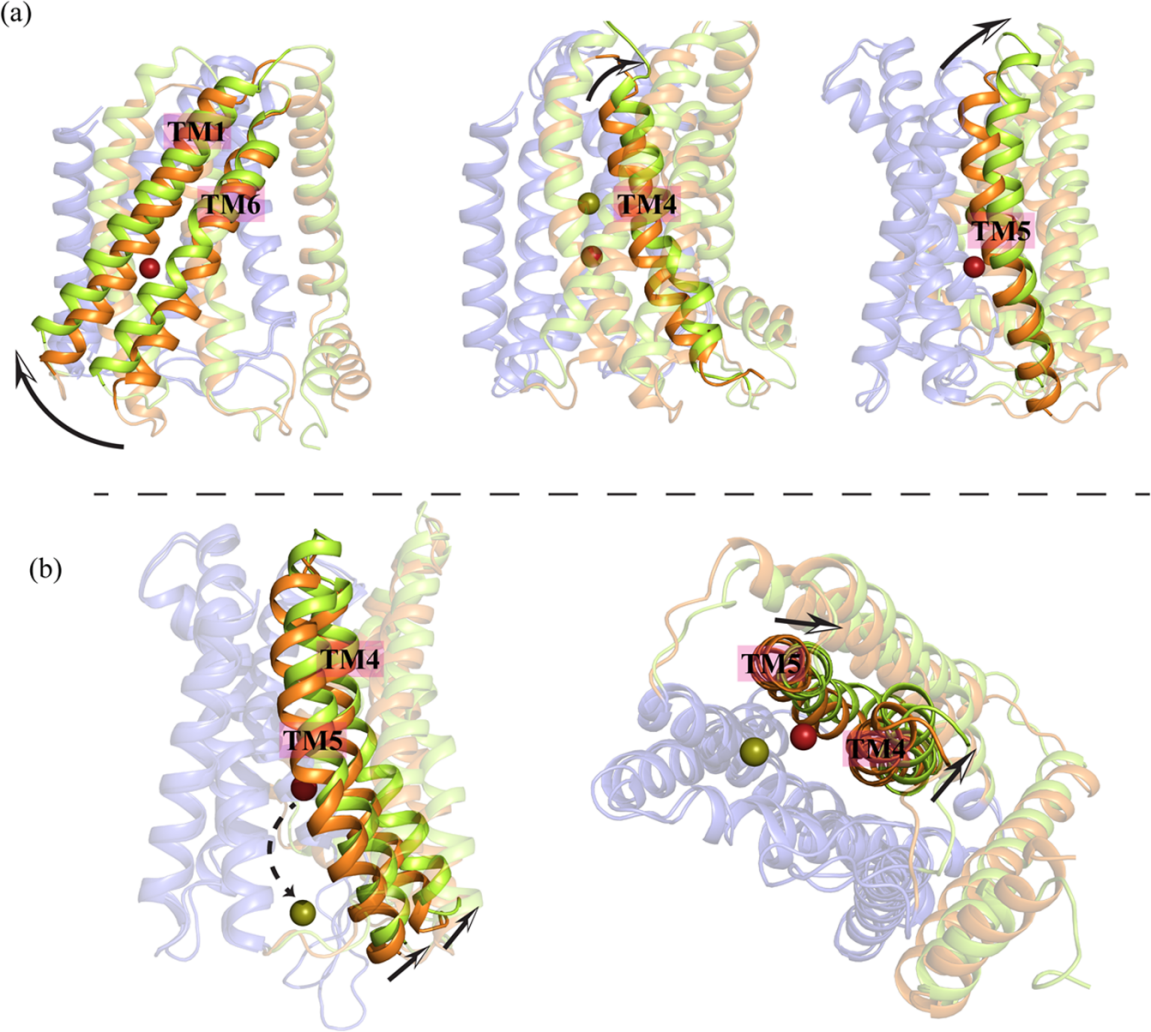
Transporter elevator motion and metal release into the cytosol observed in MTD simulations using 12-6-4 LJ parameters. (a) The elevator motion mechanism in systems with 12-6-4 LJ parameters when the metal ion moves in the reverse direction. TM1 and TM6 swing upward and to the left, while the periplasmic face of TM4 and TM5 move to the right, away from the main tunnel. (b) As the metal ion is released into the cytoplasm, the cytoplasmic portions of TM4 and TM5 move away from the main tunnel with a slight upward shift to facilitate the metal ion release.

However, the movement of TM4 and TM5 was not as significant as in the systems where 12-6-4 LJ parameters were used to model the transition metal-His/Asp/Glu side chain interactions (Figure 3a).

During this process simulated using 12-6-4, the scaffold domain remained almost static compared to the transport domain, which aligns with an experimentally validated outward-facing conformation model^33^ (The purple portion of the protein in Figure 2).

In contrast to movement toward the periplasm, three replicas out of the 24 MTD 12-6 simulations showed metal release into the cytoplasm, with the cytoplasmic parts of TM5 creating enough space for the metal ion to be released into the cytoplasm (Figure 2b). However, in most MTD replicas, the metal showed a tendency to remain at or near the M1 or M2 binding site (Table 1), despite the application of a bias potential intended to push it outside of the binding site. One possible reason for this observation could be the overestimation of the metal ion interactions with the negatively charged residues at the binding site in the standard 12-6 LJ model. It has been previously shown that the standard 12-6 LJ model cannot accurately reproduce the experimental free energy between different metal ions and negatively charged residues.^13^

Remarkably, we observed the elevator mechanism more frequently (four out of 16 replicas) in systems with 12-6-4 LJ parameters compared to those with 12-6 LJ parameters (Table 1) for transition metal-His/Asp/Glu side chain interactions. In these simulations, the metal ion moved in the reverse direction while the scaffold domain remained nearly static. The TM1 and TM6 helices in the transport domain moved upward and displayed a leftward swing (Figure 3a).

As the metal ion approached the periplasmic regions of TM4 and TM5, the periplasmic face of these helices shifted away from the main tunnel (Figure 3a). This shift, which was more pronounced for TM5 in 12-6-4 LJ systems (Compare Figures 2a and 3a), created enough space for the metal ion to be released into the periplasm. These findings support the proposed elevator mechanism^32^ and provide valuable insights into the outward-facing state of the transporter, which remains experimentally unresolved. The results also enhance our understanding of the transmembrane helices involved in the elevator mechanism and metal ion translocation.^31^

Most MTD simulation replicas using the 12-6-4 LJ model for transition metal-His/Asp/Glu side chain interactions indicated metal release into the cytoplasm (Table 1). The results revealed that when the metal ion is released into the cytoplasm, the cytoplasmic face of both TM4 and TM5 move away from the main tunnel with a slight upward shift, creating enough room for the metal ion to be released into the cytoplasm (Figure 3b). In systems with 12-6 LJ parameters, however, TM4 did not show such significant movement (Figure 2b).

**Figure 4:**
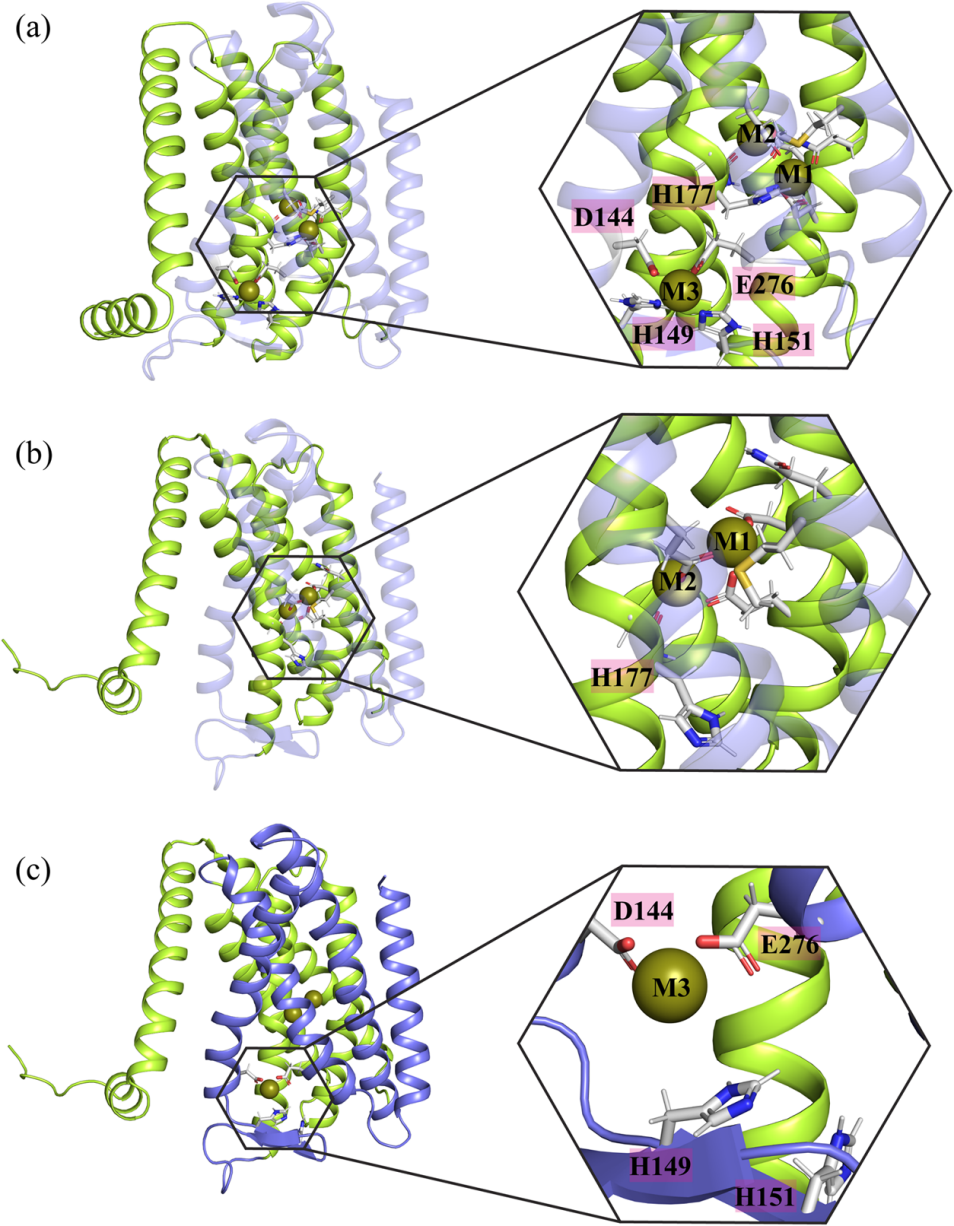
Shows the residues that coordinate with the metal ion at M1 and M3 in MTD systems using 12-6 LJ parameters. (a) The initial structure of the protein, showing the H177 residue coordinating with the metal ion at M1 in the 12-6 LJ system. (b) H177 loses its interactions with zinc at M1 after a few nanoseconds. (c) The H149 and H151 residues, crucial for coordinating with the metal ion at M3, lose their interactions with zinc as the simulation proceeds.

Overall, a comparison of Figures 2 and 4 indicates that the 12-6 LJ and 12-6-4 LJ parameter sets for transition metal-His/Asp/Glu side chain interactions yield consistent results with respect to conformational changes. The results demonstrated that when the metal ion was transported toward the periplasm, the transport domain of the protein adopted an outward-facing conformation (Figures 2a and 3a). This conformational change facilitated the release of the metal ion into the periplasm via the elevator motion mechanism.

### Key Residues in Metal Ion Transport

A detailed investigation of the residues involved in metal ion coordination indicated that H177 loses its interaction with the metal ion as the simulation progresses (Figure 4a,b).

This residue is crucial for the movement of metal from the M1 binding site to the cytoplasm by forming the M1′ binding site together with E276^32^ (The M1′ binding site is considered a transient metal binding site formed by H177 and E276 during metal ion release). These results suggest that the standard 12-6 LJ model underestimates the interaction of the zinc metal ion with histidine. This is in agreement with previous studies have shown that the 12-6 LJ model underestimates interactions between the nitrogen atom of the imidazole side chain of histidine residues and various divalent metal ions.^12^

We also examined the interactions of the M3 binding site residues with the metal ion in the simulation setups where the M3 binding site was occupied by the metal ion (second and fourth setups). The results indicated that H149 and H151, which are essential residues for coordinating the metal ion at M3, lose their interactions with the metal ion (Figure 4c). However, the loop remains close to M3 and continues to interact with the metal ion through D144 and E276. The M3 site retains the metal ion due to the strong (overestimated) interactions with the negatively charged residues D144 and E276 (Figure 4c).

These findings suggest that, although metal release into either the cytoplasm or periplasm was rarely observed, the metal ion pathway explored by the MTD simulation and the estimated FES might not be accurate using 12-6 LJ parameters for transition metal-His/Asp/Glu side chain interactions. Therefore, we employed a new model combining the WT volume-based MTD method with the 12-6-4 LJ model for transition metal-His/Asp/Glu side chain interactions to determine if improved results could be obtained compared to the standard 12-6 LJ model. Full details of this work have been reported elsewhere,^31^ so our focus here is on the comparison of the 12-6 model versus the 12-6-4. In four different simulation setups (mentioned above) using 12-6-4 LJ model with at least three simulation replica each (totaling 16 simulations), we observed the metal ion remaining at the BMC binding site in only two simulation replicas. This allowed the WT volume-based MTD method to explore more areas and potential metal ion escape pathways in the transporter. In other words, most simulation replica using the 12-6-4 LJ model showed the metal ion being released into the cytoplasm or periplasm.

The predominant presence of acidic residues at the BMC binding site generates a negatively charged cavity. With inaccurate parameters, such as those in the 12-6 LJ model for metal ion-acidic residue interactions, the metal ion forms strong interactions with surrounding negatively charged residues. Previous studies have shown that the 12-6 LJ model overestimates these interactions.^13^ Even when applying the same bias potential with a constant force as in the 12-6-4 LJ model, it is difficult to overcome this resistance, causing the metal ion to remain at or around the M1/M2 binding site. However, by adjusting the parameters to more accurately estimate interactions between negatively charged residues and the metal ion using the 12-6-4 LJ model, the metal ion can leave the BMC binding site and explore more potential pathways for release into either the cytoplasm or periplasm.

**Figure 5:**
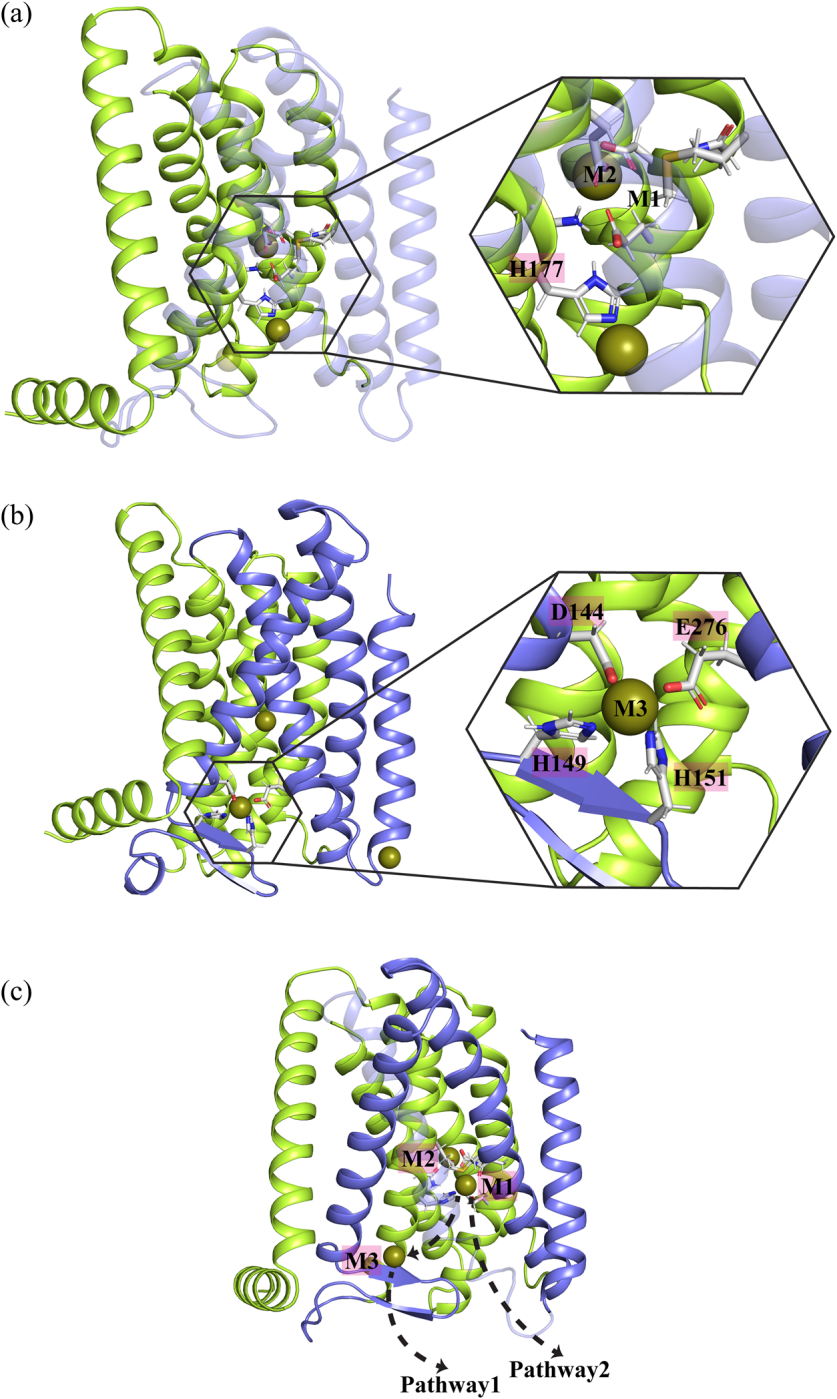
Shows the residues that coordinate with the metal ion at M1 and M3 in MTD systems using 12-6-4 LJ parameters. (a) and (b) H177 not only effectively interacts with the metal ion in systems with 12-6-4 LJ parameters but also assists with metal transport toward the cytoplasm or M3 from the M1 binding site. H149 and H151, which are important for forming the M3 binding site for the metal ion, continue to interact with zinc throughout the simulations. (c) The two proposed pathways for metal release into the cytoplasm.

We analyzed the interactions between metal ion and the crucial H177 residue, as well as the residues at the M3 binding site in the 12-6-4 LJ systems. The results showed that the H177 residue not only maintains its interactions with the metal ion but also facilitates its transport toward the M3 binding site or the cytoplasm in systems where the metal ion is released into the cytoplasm (Figure 5a).

Furthermore, in contrast to the systems with standard 12-6 LJ parameters for transition metal-His/Asp/Glu side chain interactions, the MTD simulations with 12-6-4 LJ parameters showed that all residues involved in coordinating the metal ion at M3 maintained their interactions throughout the simulation (Figure 5b).

**Figure 6:**
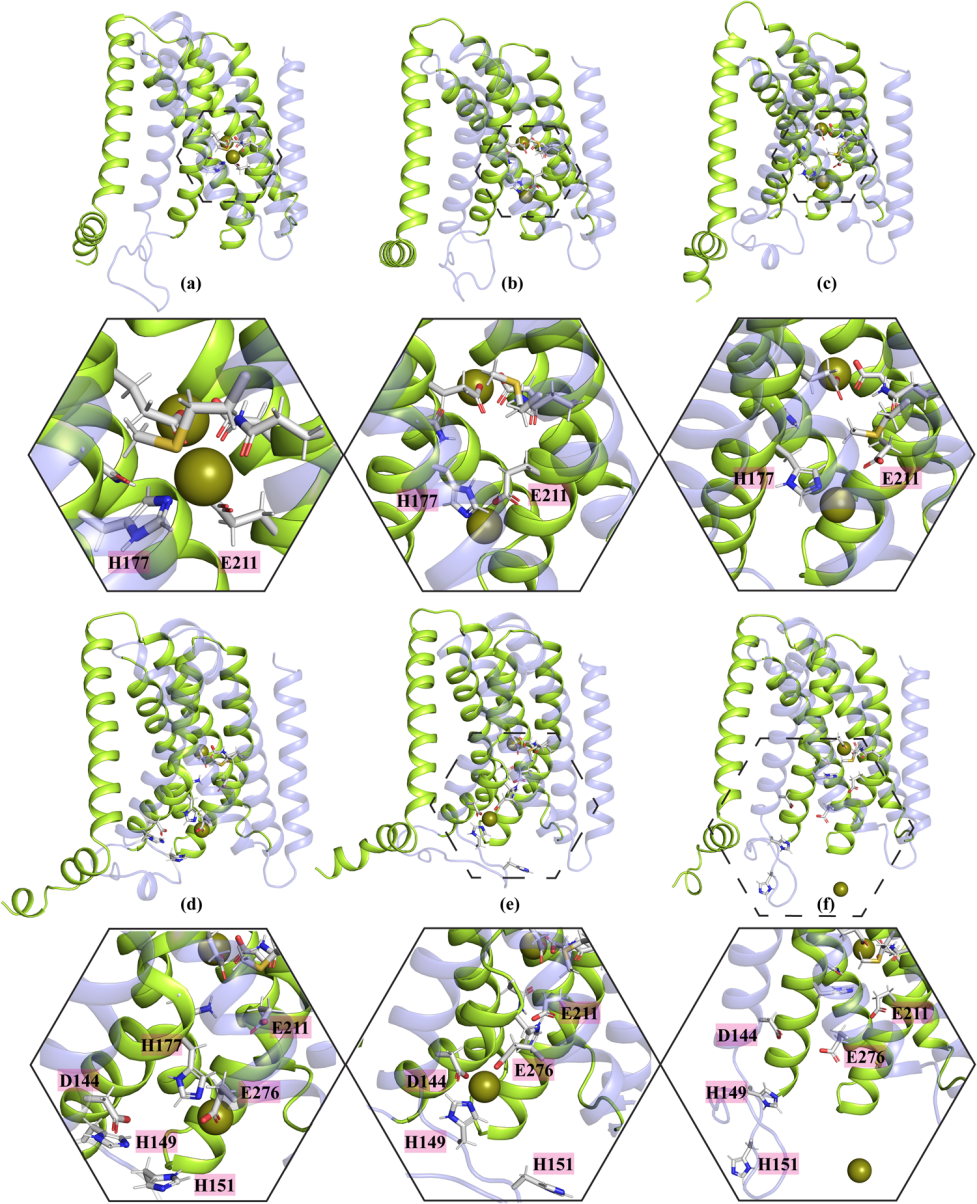
A step-by-step representation of metal transport from the M1 binding site toward the M3 binding site or the cytoplasm in systems with 12-6-4 LJ parameters, highlighting the important residues involved in this process. The hexagons with solid lines show a zoomed-in view of the areas highlighted with dashed-line hexagons in each panel.

A detailed investigation of the potential metal release pathways into the cytoplasm revealed two routes. The metal ion is either released by moving toward the M3 binding site in systems with an unoccupied M3 (Pathway 1) or through the main tunnel in systems where the M3 binding site is occupied (Pathway 2) (Figure 5c). Analysis of residue interactions demonstrated that the metal ion initially interacts with H177 and E211, then transfers to M1′ (H177-E276) before being directly released into the cytoplasm (Figure 6) or indirectly released into the cytoplasm via transfer to the M3 binding site. However, H151, one of the four residues involved in the M3 binding site (D144, E276, H149, H151), did not coordinate with the metal ion upon its arrival at the binding site (Figure 6).

**Figure 7:**
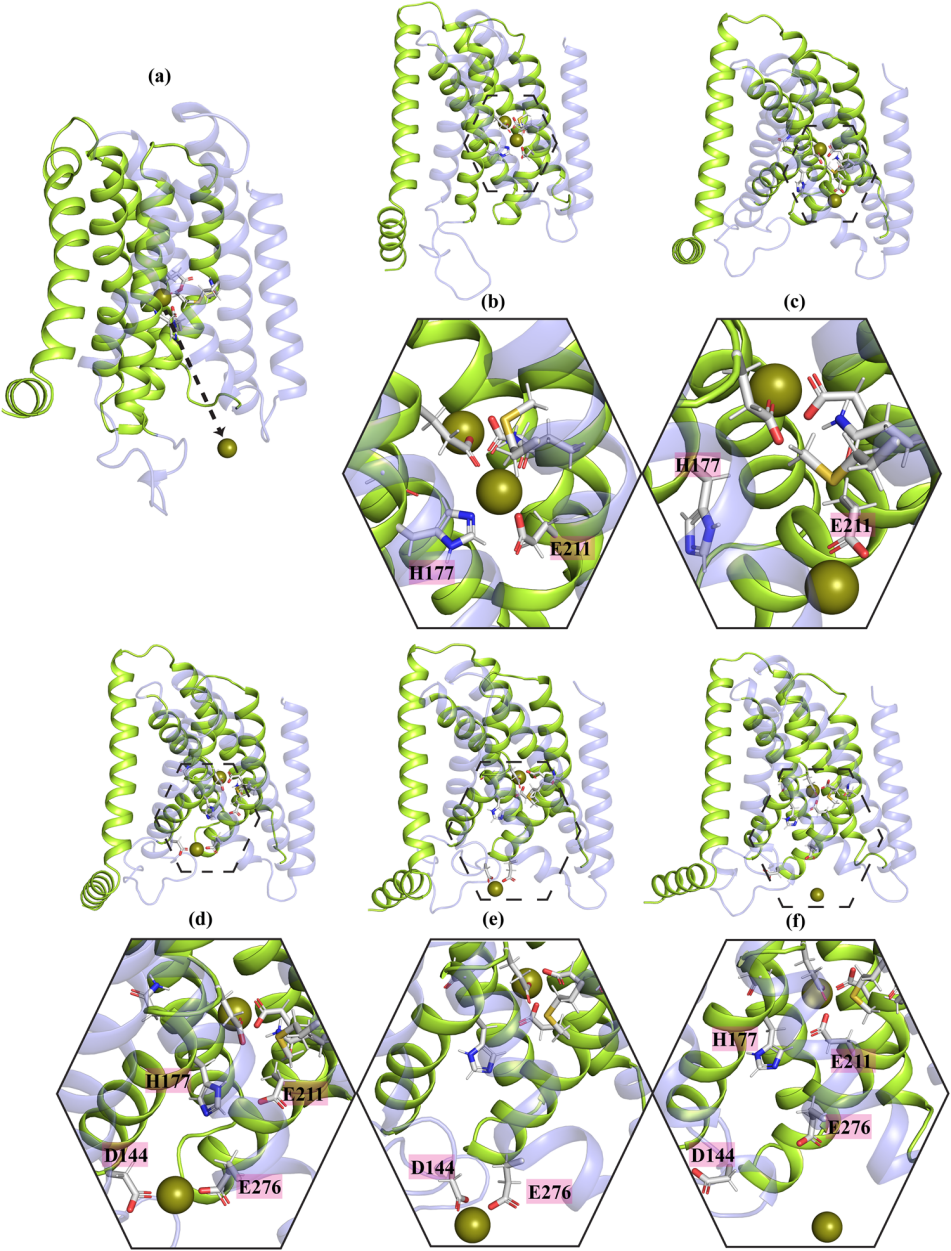
(a) The sudden release of the metal ion from M1 to the cytoplasm in MTD simulation systems with 12-6 LJ parameters. (b) to (f) A detailed step-by-step illustration of metal transport from the M1 binding site to either the M3 binding site or the cytoplasm in systems with 12-6 LJ parameters, highlighting the residues involved. Solid-line hexagons provide a zoomed-in view of the areas indicated by dashed-line hexagons in each panel.

In all 12-6 LJ models for transition metal-His/Asp/Glu side chain interactions, the histidine residues at M1 or M3 lost their interactions with the metal ion after a few nanoseconds (Figure 3). Additionally, one replica of the second setup using the 12-6 LJ model showed that the metal ion is released into the cytoplasm without the involvement of the important residues of the main tunnel (Figure 7a).

A similar phenomenon was observed in one replica of the first setup, while in another replica of the same setup, H177 was not involved, and the metal moved from M1 to an area between D144 and E276 before being released into cytoplasm (Figure 7b–f). As previously mentioned, the standard 12-6 LJ model underestimates the interactions between histidine residues and various divalent metal ions, including zinc.^12^ This underestimation may explain the release of metal ions into the cytoplasm without the involvement of histidine residues at the M3 site, which are known to be important for metal ion coordination at the M3 binding site.

Our results indicated that 12-6-4 LJ parameter sets for transition metal-His/Asp/Glu side chain interactions improves the metal ion interactions with surrounding residues, thus using enhanced sampling method like WT volume-based MTD produces results that align well with experimental observations. In contrast, using 12-6 LJ parameter sets failed to reproduce previous experimental observations due to inaccurate estimation of the metal ion-residue interactions within the transporter.

### Comparative Analysis of the Free Energy Surface

**Figure 8:**
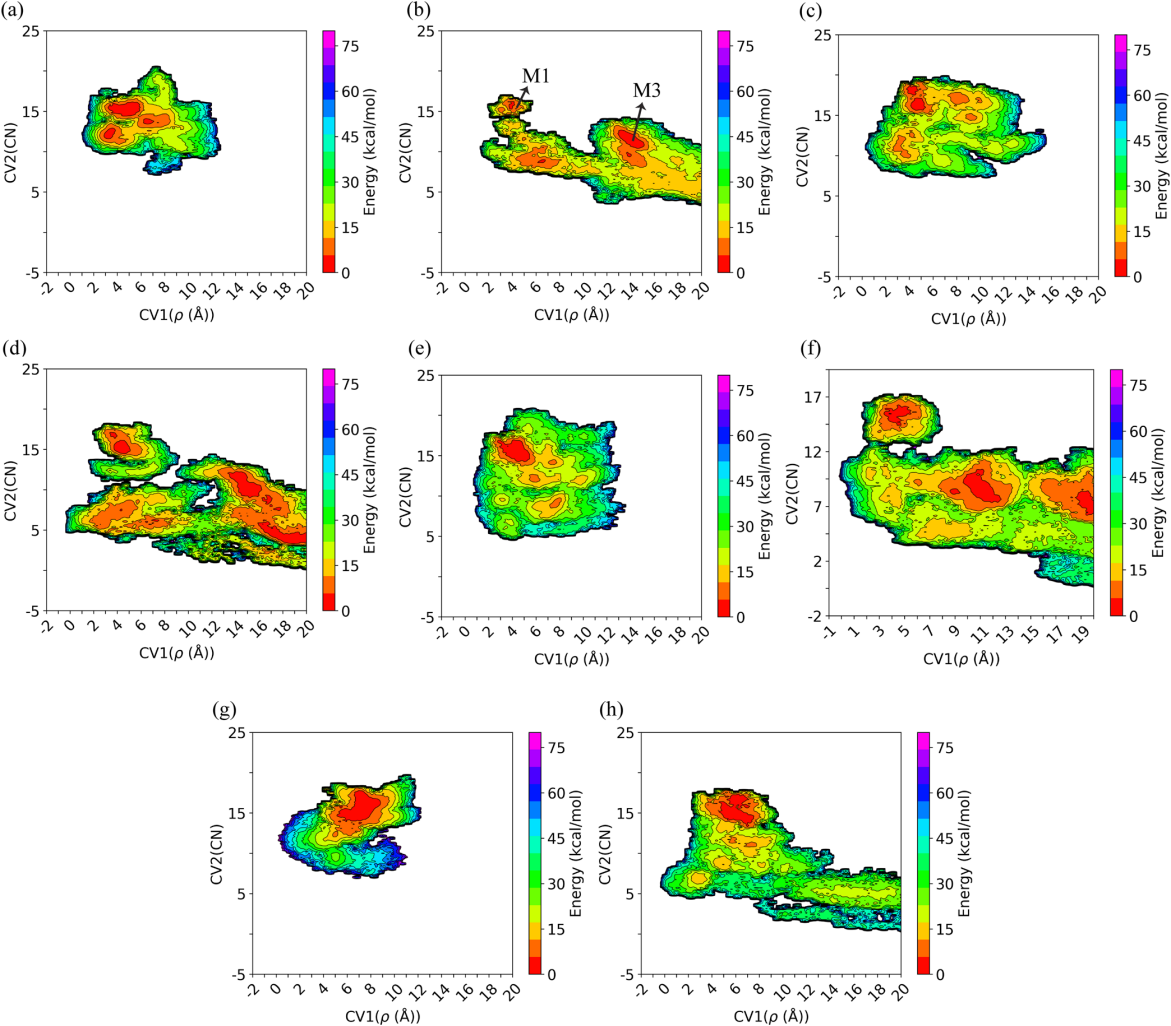
Two-dimensional FES map of the simulation systems reconstructed based on the metal ion coordination number (CV2) and the Euclidean distance of the metal ion from the sphere’s center of mass (CV1). As shown in panel (a), the metastable states of the system occur when the metal ion is near the BMC binding site. Panel (a) indicates the system with 12-6 LJ parameters, while the system with 12-6-4 LJ parameters (b) displays a greater number of local minima corresponding to both the BMC and M3 binding sites. This suggests that the metal ion explores a larger area within the defined spherical region. A comparison between panels (c) and (d), (e) and (f), and (g) and (h) indicates that the 12-6-4 LJ parameters (the (d), (f), and (h) panels) allow the metal ion to move more freely within the spherical region, showing more local minima. In contrast, using standard 12-6 LJ parameters confines the metal ion to the BMC binding site or its vicinity, due to the inaccurate estimation of interactions between negatively charged residues and histidine with the metal ion. Panels (a)/(b), (c)/(d), (e)/(f), and (g)/(h) each correspond to one simulation setup, with each pair representing the first replica of the first through fourth simulation setups, respectively.

To obtain the two-dimensional (2D) FES map of each simulation system during metal ion transport, we calculated the FES of all systems using two variables including the Euclidean distance of the metal ion from the sphere center (CV1, ρ) and the metal coordination number (CV2, CN) (Figure 8).

In the initial simulation setup using the standard 12-6 LJ model for transition metal-His/Asp/Glu side chain interactions, the M1 and M2 binding sites are occupied by the metal ion while the M3 binding site remains vacant with IL2 unfolded. In this configuration, only one metastable state (around 4–6 Å) was observed, corresponding to the BMC binding site (Figures 8a and S1a–S1e). Based on the 2D free energy plots presented in Figures 8a and S1, the metal ion explores the vicinity of the M1 binding site but remains confined to this site, except in two replicas where the metal ion is released into the cytoplasm (the fifth and sixth replicas, Figures S(d) and S(e)). In these instances, the system still exhibits only one local minimum associated with the M1 binding site. As shown in Figure S(d), there is a gap between the two explored areas, indicating the abrupt release of the metal ion without interaction with specific residues within the main metal release tunnel (Figure 7a). On the contrary, in simulations with 12-6-4 LJ parameters, the metal ion transitions to a second local minimum, M3, where D144, H149, and E276 coordinate the metal ion before its release (Figures 6, S1f, and S1g). Thus, the first and second local minima shown in the 2D free energy plot correspond to the BMC and M3 binding sites, respectively. Furthermore, as illustrated in Figure 8b, compared to the 12-6 LJ model (Figure 8a), the 12-6-4 LJ parameter set allows the metal ion to explore more pathways and move freely within the transporter.

In the second simulation setup using 12-6 LJ parameters, results identical to those of the first setup were observed (Figures 8c and S2). In most simulation replicas, the metal ion remained stationary at the center of the transporter near the M1 binding site, indicating a stable configuration. Thus, a single local minimum associated with the BMC binding site was identified (Figure S2). However, in the fourth and fifth replicas of this simulation setup (Figure S2c,d), the metal ion was observed to be released either into the cytoplasm or periplasm. Figure S2c illustrates a gap in the 2D free energy plot of the fourth replica, indicating the abrupt release of the metal ion into the cytoplasm without engaging with the residues comprising the M1′ binding site (Figure 7a). Unlike most MTD simulations with 12-6 LJ parameters, simulations using 12-6-4 LJ parameters indicated that the metal ion generally does not remain at the BMC binding site but instead diffuses into the cytoplasm or periplasm. This results in a broader exploration of the metal ion within the transporter’s main tunnel and facilitates the identification of additional potential pathways local minima (Figures 8d) and pathways for metal ion release (Figure S3). This behavior is attributed the correct modeling of the interactions with acidic residues and the important H177 residue involved in metal transport.

In the third simulation setup, where the M1 and M2 binding sites were occupied by the metal ion and the M3 binding site remained empty with IL2 folded, the results from the 12-6 LJ model indicated that the metal ion tended to remain around the M1 binding site (Figure 8e). The applied bias potential could not completely overcome the resistance caused by the overestimated interactions between acidic residues and the metal ion at the BMC binding site. And the metal cannot move toward either the cytosol or periplasm in most instances. As a result, only a limited area around the M1 binding site was explored, restricting the metal exploration of potential pathways inside the transporter (Figure S4). In one replica (the fourth replica, Figure S4c), however, the metal freely moved toward the cytoplasm but remained confined within the transporter without being released. On the other hand, in simulations employing 12-6-4 LJ parameters, the metal was released into either the cytoplasm or periplasm in all replicas (Figures 8f and S5). Moreover, in two out of three replicas (Figures 8f and S5a), multiple local minima suggested that the metal ion explored diverse pathways within the transporter.

In the last simulation setup, M2 was empty while M1 and M3 were occupied by the metal ion, and IL2 was folded. Similar outcomes were observed as in other setups using 12-6 LJ parameters, suggesting that with these parameters, the metal ion tends to remain at the BMS binding site, limiting alternative pathways (Figures 8g and S6). Alternatively, with 12-6-4 LJ parameters, only one out of four replicas retained the metal ion at the M1 binding site (Figure S7b). In the other replicas, the metal ion was either released into the cytoplasm or periplasm, with the transporter indicating elevator motion upon release into the periplasm in two replicas (Figures 8h and S7).

To compare the two nonbonded LJ potentials quantitatively, we designed a more conservative WT-MTD setup. This approach limited the exploration of the metal ion’s release into the cytoplasm, focusing instead on interactions within the first 5 Å from the metal site toward the cytosol by constraining the CV exploration. Using this methodology, we achieved a converged free energy profile within 500 ns of biased simulation for both 12-6 and 12-6-4 LJ nonbonded models (see Figure S8).

The results are presented in a two-dimensional (2D) FES map using the metal ion position from the transporter center of mass projected along the *z* axis (CV1, z) and the metal coordination number (CV2, CN) as defined previously. They show that the 12-6 LJ parameters exhibit minima at the M1 and M2 sites (∼–1 Å on the FES map) with larger coordination numbers compared to the 12-6-4 LJ parameters. As previously noted, this suggests that the standard 12-6 LJ parameters may overestimate the interactions between the metal ion and the surrounding residues in this region. Across all presented FES maps, the pathway from the M1/M2 sites to the M3 site (∼-5 Å) is notably facilitated when using the 12-6-4 LJ parameters compared to the 12-6 LJ parameters. While the energetic differences can be quantified, we currently lack experimental data for direct validation of these findings.

Overall, in all simulations, including those with 12-6 LJ and 12-6-4 LJ parameters, we detected a major local minimum around the BMC binding site, where the metal tends to be trapped in such a highly negatively charged area. However, with the 12-6-4 LJ parameters, the metal ion interacts more accurately with negatively charged residues in the metal release pathway, alongside stronger interactions with important histidine residues. This enables the metal ion to depart from the BMC binding site and explore additional escape pathways and local minimum compared to standard 12-6 LJ parameter sets. This exploration may uncover additional pathways and local minima in metal release phenomena, not only in the studied protein but also in other divalent metal ion transporters.

### The Effects of Standard and Finely Tuned LJ Parameters on the Spherical Coordinates

**Figure 9:**
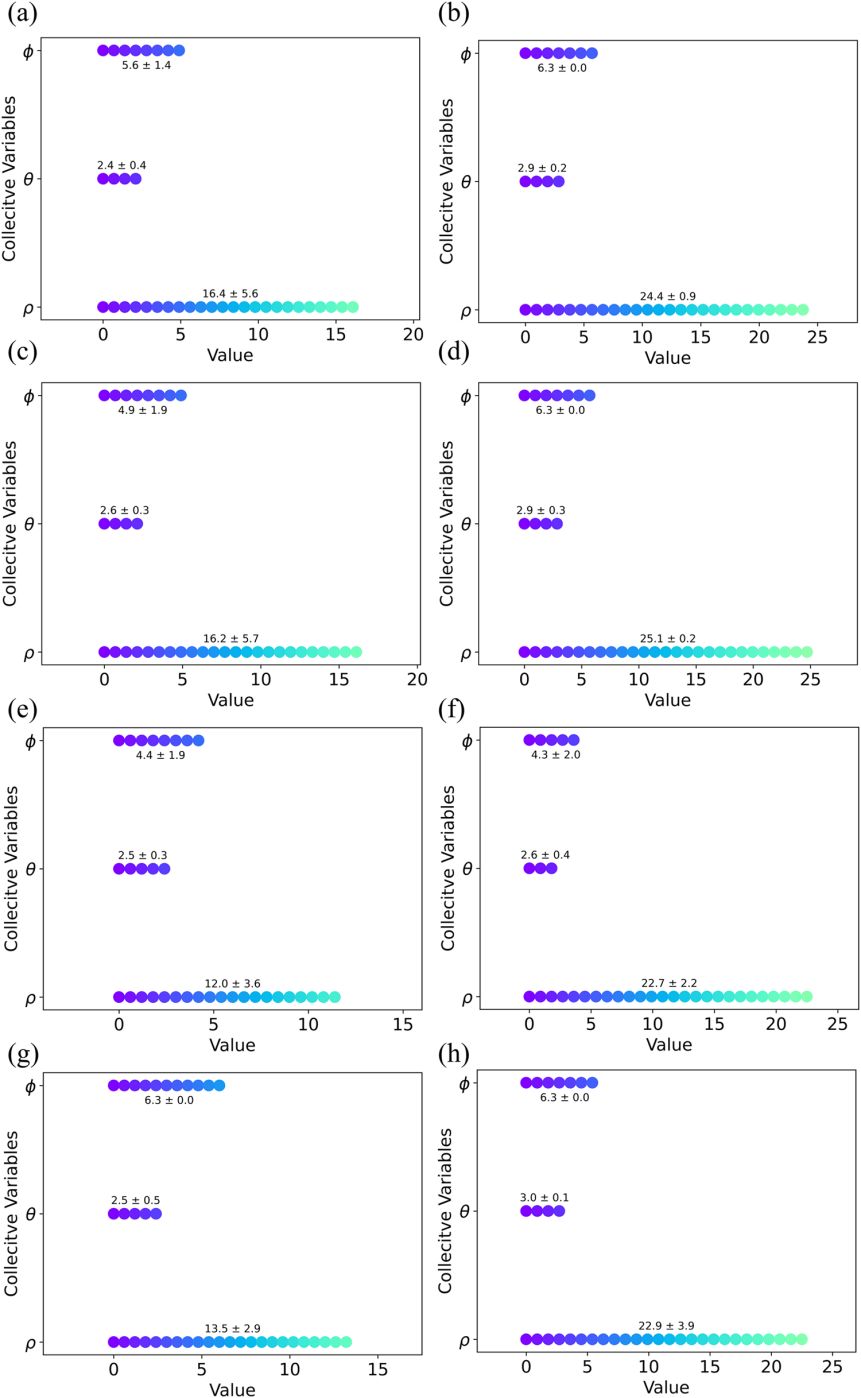
Displays the average values of the collective variables for both 12-6 and 12-6-4 LJ MTD systems. The plots in the left column (panels (a), (c), (e), and (g)) represent the 12-6 LJ systems, while the plots in the right column (panels (b), (d), (f), and (h)) represent the 12-6-4 LJ systems. In each plot, ρ, θ, and φ denote the spherical coordinates (collective variables). The first through fourth rows display the simulation setups listed in Table 1, respectively.

In this study, a spherical restraint was applied, involving a sphere centered around the helices forming the metal ion transport tunnel. This approach limits the volume accessible to the metal ion, increasing the probability of binding and unbinding processes within this confined space, as described in previous work.^10^ The CVs used within this spherical restraint are the spherical coordinates (ρ, θ, φ) of the metal ion relative to the center of mass of the helices that form the metal ion transport pathway (Figure S9). These CVs effectively capture the metal ion’s position and movement within the confined spherical volume, as described in previous work.^10,48^ Therefore, we can explore all possible escape pathways of the metal ion within the transporter’s environment. We calculated the average values of these CVs across all systems during the simulations (Figure 9).

The calculated CVs values for the first and second setups show higher φ, θ, and ρ averaged values for the systems with 12-6-4 LJ models, indicating that the metal had more freedom to move within the defined spherical restraint (Figure 9a–d). This difference is more pronounced when comparing the ρ value range of the 12-6-4 LJ systems to that of the 12-6 LJ systems, as the metal is released into the cytoplasm or periplasm more frequently. The average CV values in the third and fourth setups generally follow the same trend as the first and second simulation setups, with higher ρ value ranges (Figure 9e,h).

In general, the results indicated that with the 12-6 LJ parameter set, the metal tends to get stuck at the BMC binding site. In contrast, using the 12-6-4 LJ parameters, where the interactions between histidine/negatively charged residues and the metal ion are adjusted, allows the metal to move more freely and explore the defined area. This enables the discovery of more possible metal ion pathways and metastable states of the system during the metal ion release process.

One might imagine that by extending the simulation time length using 12-6 LJ parameters or increasing the applied force constant, we may be able to observe metal release. However, as described in previous sections, we may not capture the important residues involved in the metal release due to inaccurate metal-residue interactions using the 12-6 LJ model. Additionally, we used the same conditions for both the 12-6 LJ and 12-6-4 LJ models to identify the differences between these parameters in MTD simulations. The results showed that, under the same conditions, the 12-6-4 LJ model outperforms the 12-6 LJ model in the metal transporter systems.

## Conclusions

In summary, the results indicate that the WT-MTD simulations combined with the 12-6-4 LJ model for transition metal-His/Asp/Glu side chain interactions outperforms the regular 12-6 LJ model for these same interactions for the ZIP metal transporter. The 12-6-4 LJ model helps WT-MTD simulations in observing more frequently both the elevator mechanism and the metal release into the cytoplasm compared to the standard 12-6 LJ model. Moreover, the more accurate 12-6-4 LJ model improves the interactions between histidine/negatively charged residues involved in metal transport and the metal ion and facilitates the observation of residues implicated in metal transport, as suggested by previous experimental studies. This model, when combined with the WT-MTD simulations, allows the metal ion to explore a broader range of areas and potential release pathways during transport in shorter simulation time, while also enhancing computational efficiency. This observation is even more remarkable given the fact that the model is dominated by 12-6 LJ interactions for the majority of the system, while the transition metal-His/Asp/Glu side chain interactions are the only interactions using the 12-6-4 LJ model. Hence, a small subset of interactions significantly modifies the states sampled by the entire protein leading to a more frequent observation of the transport of the transition metal ion. WT-MTD combined with the 12-6-4 LJ model for transition metal-His/Asp/Glu side chain interactions can find potential applications in various metalloproteins and metal transporters and could contribute to advancement in drug discovery focusing transition metal ion channels/transporters and on deepening our understanding of transition metal ion transport mechanisms.

## Supporting Information Available

The two-dimensional FES maps from the remaining MTD simulations, along with a representation of the collective variables that are used to specify the position of the metal ion during the MTD simulations (PDF)
