# Supplementary material for: Ion-Induced Dipole Interactions Matter in Metadynamics Simulation of Transition Metal Ion Transporters
Source: J Chem Theory Comput. 2025 Apr 3;21(8):4221–35. doi: 10.1021/acs.jctc.4c01535 (PMC12020355; doi:10.1021/acs.jctc.4c01535)
Supplement: Supplementary file 1 — ct4c01535_si_001.pdf [file ct4c01535_si_001.pdf]

Ion-induced Dipole Interactions Matter in Metadynamics Simulation of Transition Metal Ion Transporters

Majid Jafari,[1] Luca Sagresti,[2,3] Jian Hu,[1,4] Kenneth M. Merz, Jr.[1,4,*]

[1]Department of Biochemistry & Molecular Biology, Michigan State University, MI 48824, United States

[2]Scuola Normale Superiore, Piazza dei Cavalieri 7, I-56126 Pisa, Italy and CSGI

[3]Istituto Nazionale di Fisica Nucleare (INFN) sezione di Pisa, Largo Bruno Pontecorvo 3, 56127 Pisa, Italy

[4]Department of Chemistry, Michigan State University, MI 48824, United States

[*]*Corresponding Author Email:* Kenneth M. Merz, Jr, merz@chemistry.msu.edu

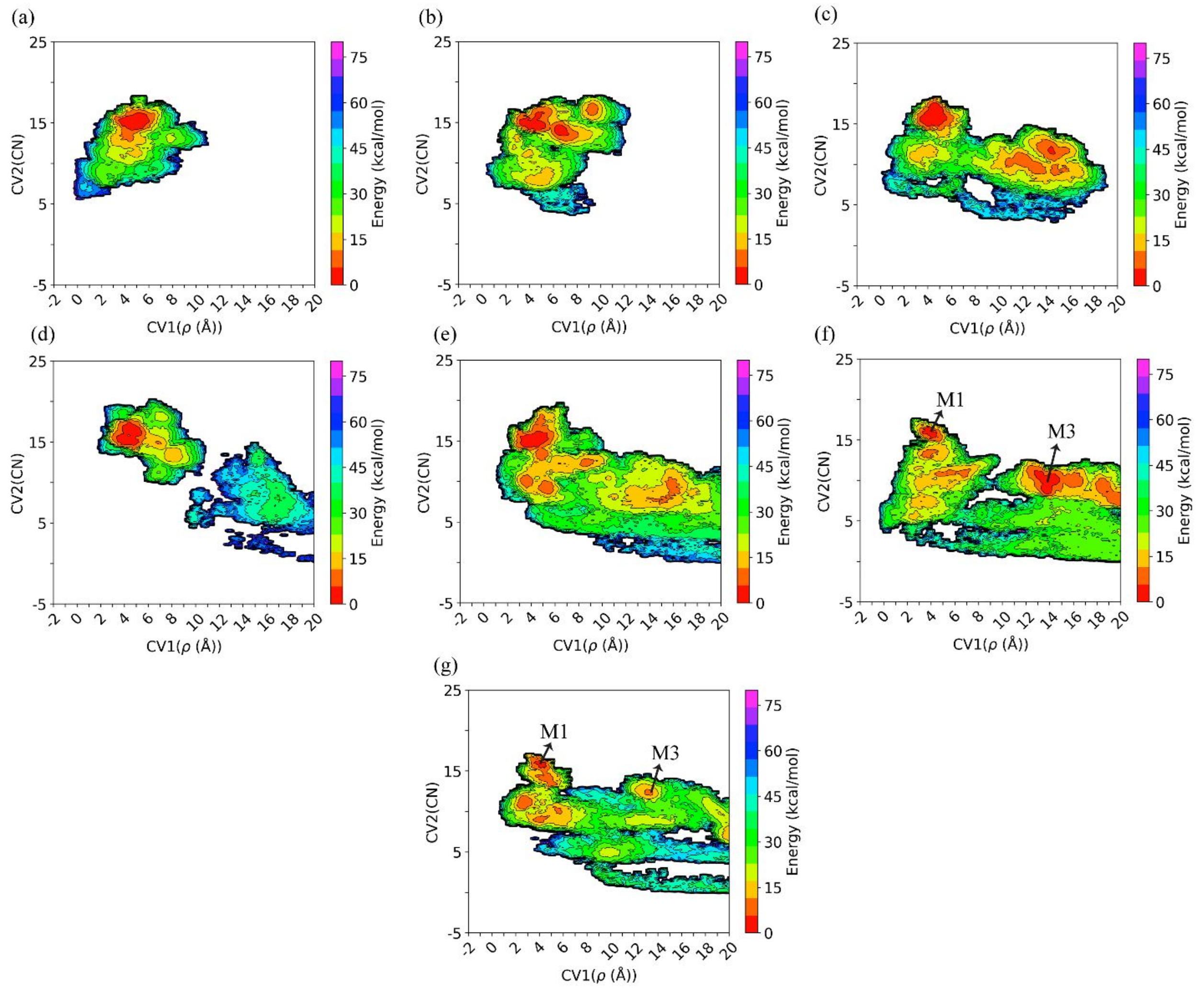

Figure S1. Panels (a) to (e) show the two-dimensional (2D) free energy surface (FES) maps from the remaining metadynamics (MTD) simulations of the first setup using 12-6 LJ parameters, which are not shown in Figure 8. Panels (f) and (g) display the 2D FES maps for the same setup using 12-6-4 LJ parameters, with (f) and (g) indicating the second and third replicas, respectively. M1 and M3 mark the metal ion M1 and M3 binding sites. CV2 represents the metal ion coordination number, and ρ is the metal ion distance from the sphere's center of mass. Panels (a) to (e) correspond to the second through sixth simulation replicas, respectively.

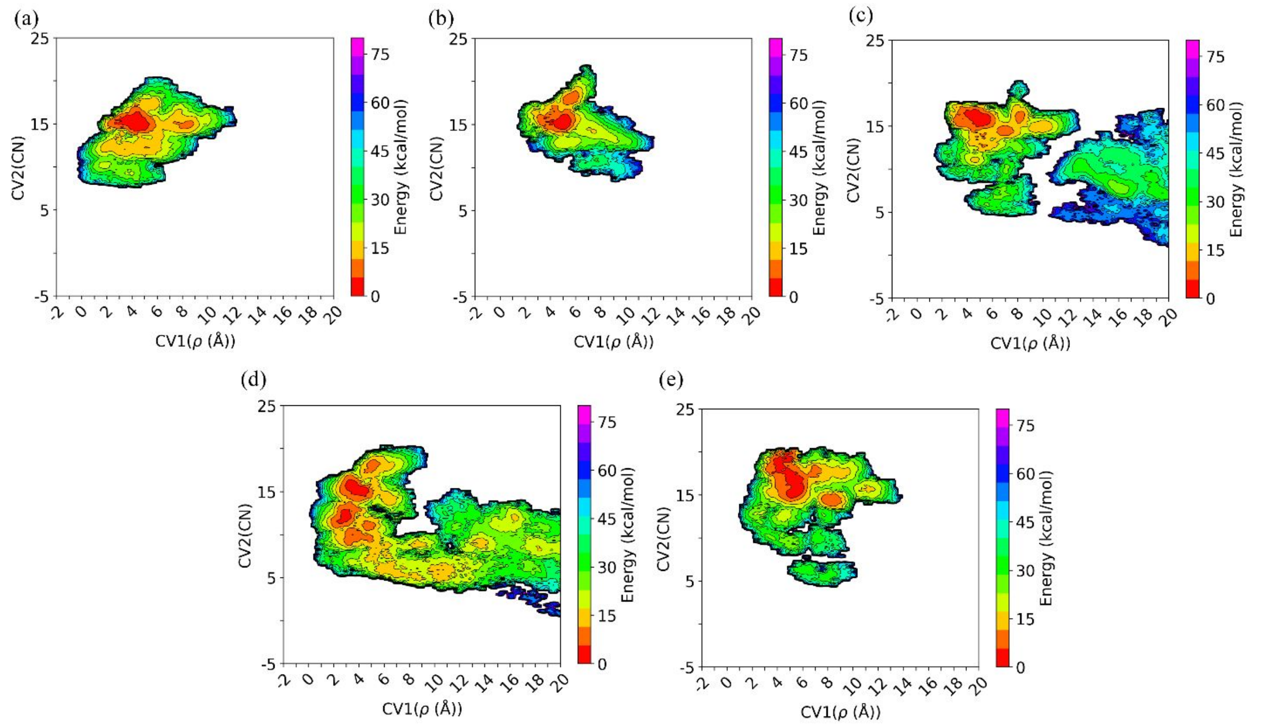

Figure S2. Shows the 2D FES maps from the remaining simulation replicas of the second setup using 12-6 LJ parameters, which are not shown in Figure 8. CV2 represents the metal ion coordination number, and ρ is the metal ion distance from the sphere's center of mass. Panels (a) to (e) correspond to the second through sixth simulation replicas, respectively.

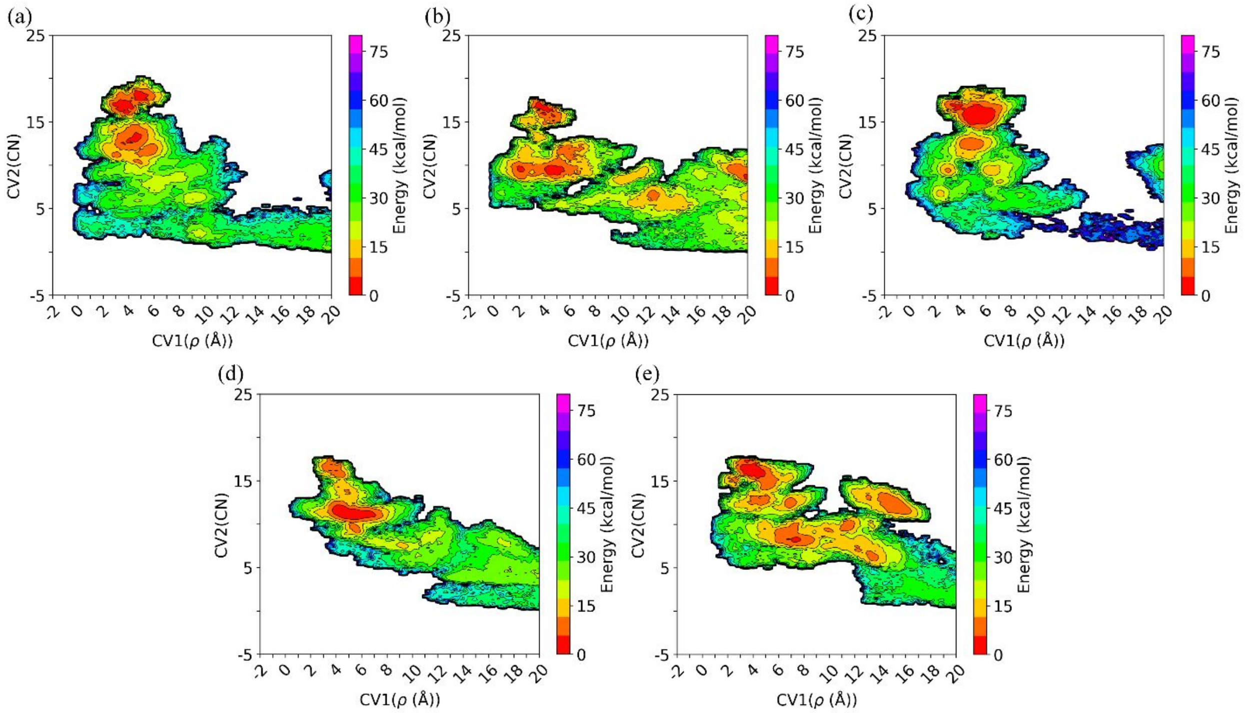

Figure S3. Represents the 2D FES maps from the remaining simulation replicas of the second setup using 12-6-4 LJ parameters, which are not shown in Figure 8. CV2 represents the metal ion coordination number, and ρ is the metal ion distance from the sphere's center of mass. Panels (a) to (e) correspond to the second through sixth simulation replicas, respectively.

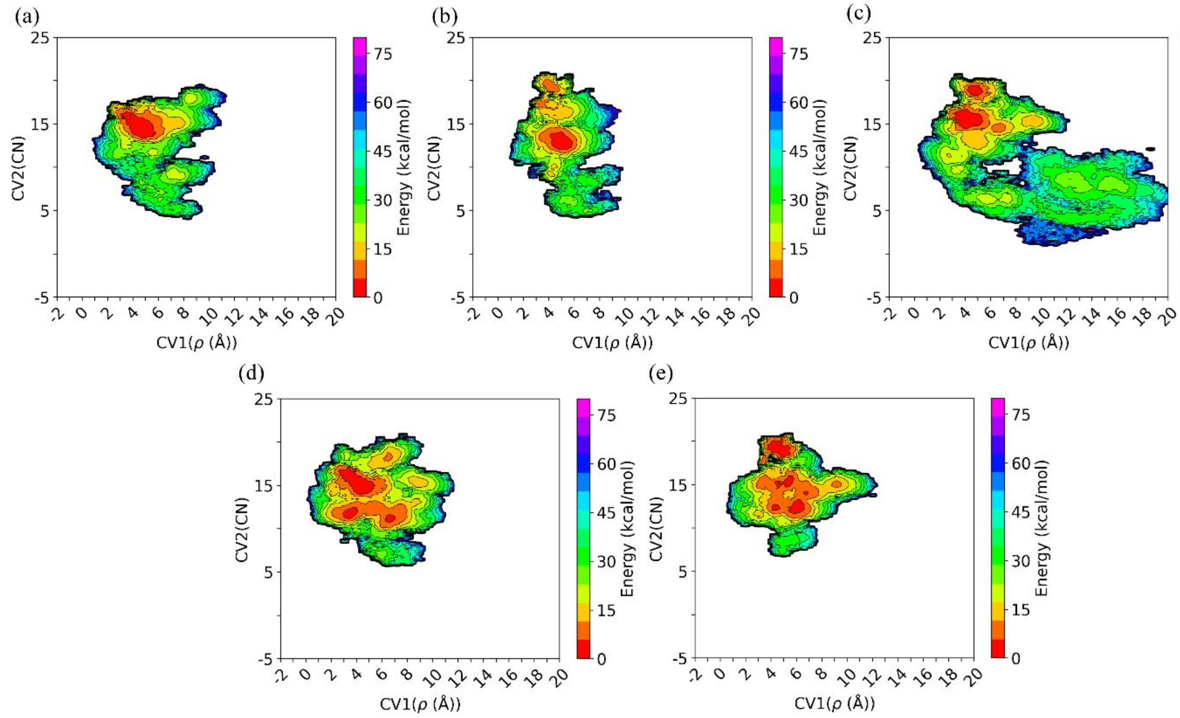

Figure S4. The 2D FES maps from the remaining simulation replicas of the third setup using 12-6 LJ parameters, which are not shown in Figure 8. CV2 represents the metal ion coordination number, and ρ is the metal ion distance from the sphere's center of mass. Panels (a) to (e) correspond to the second through sixth simulation replicas, respectively.

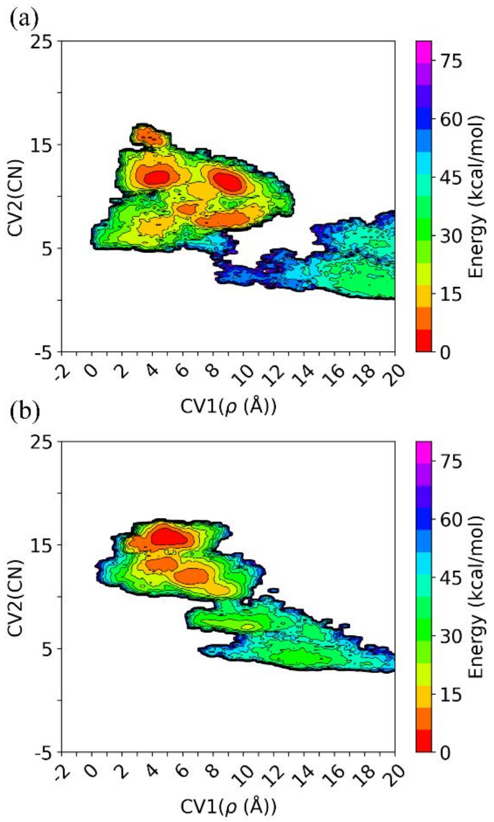

FigureS5. The 2D FES maps from the remaining simulation replicas of the third setup using 12-6-4 LJ parameters, which are not shown in Figure 8. CV2 represents the metal ion coordination number, and ρ is the metal ion distance from the sphere's center of mass. Panels (a) to (b) correspond to the second and third simulation replicas, respectively.

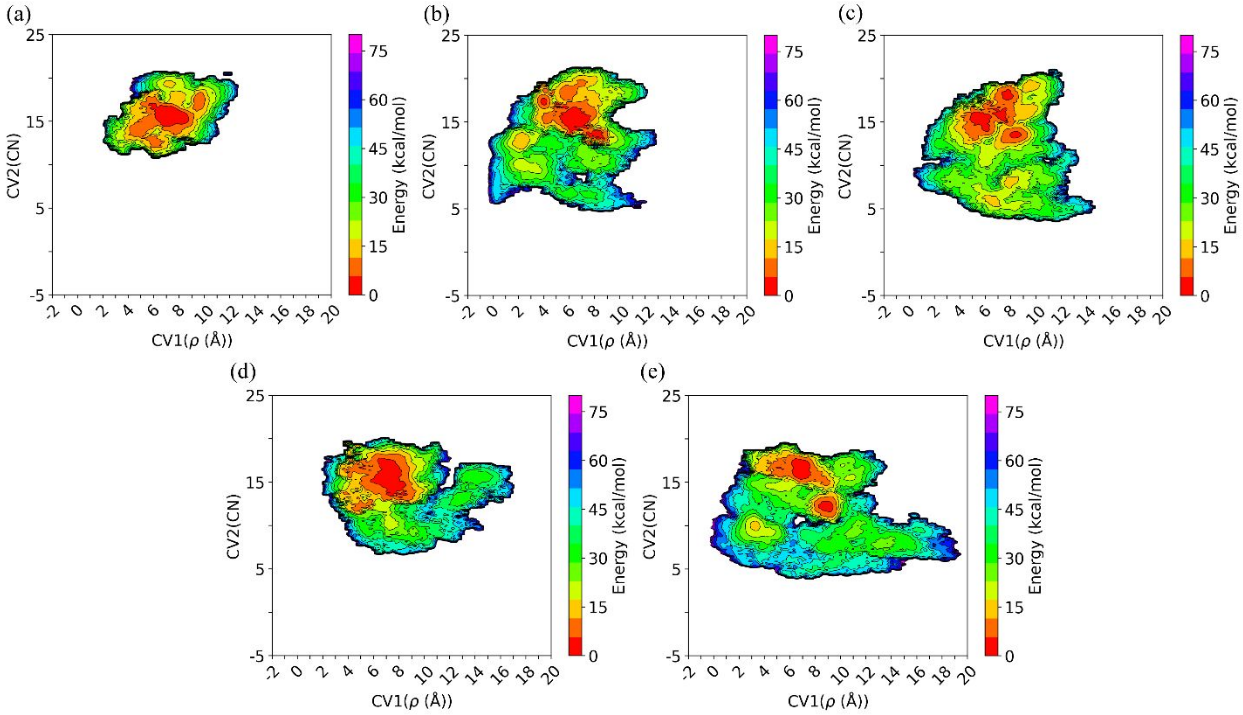

Figure S6. Illustrates the 2D FES maps from the remaining simulation replicas of the fourth setup using 12-6 LJ parameters, which are not shown in Figure 8. CV2 represents the metal ion coordination number, and ρ is the metal ion distance from the sphere's center of mass. Panels (a) to (e) correspond to the second through sixth simulation replicas, respectively.

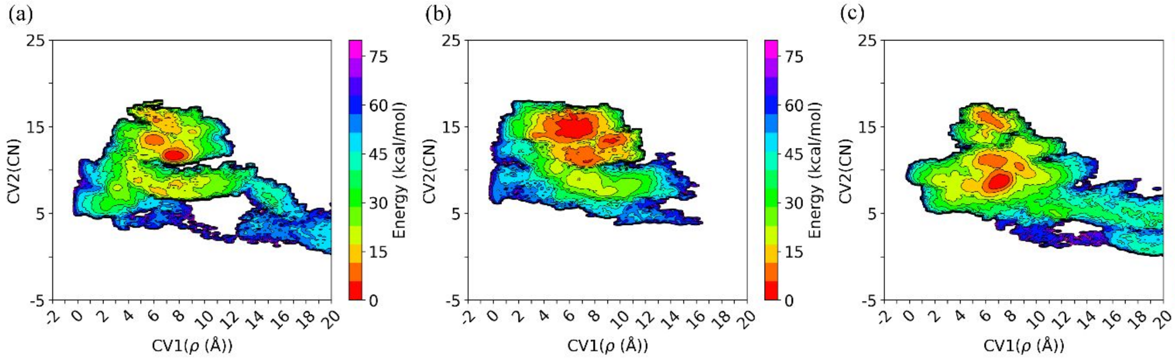

Figure S7. Illustrates the 2D FES maps from the remaining simulation replicas of the fourth setup using 12-6-4 LJ parameters, which are not shown in Figure 8. CV2 represents the metal ion coordination number, and ρ is the metal ion distance from the sphere's center of mass. Panels (a) to (c) correspond to the second through fourth simulation replicas, respectively.

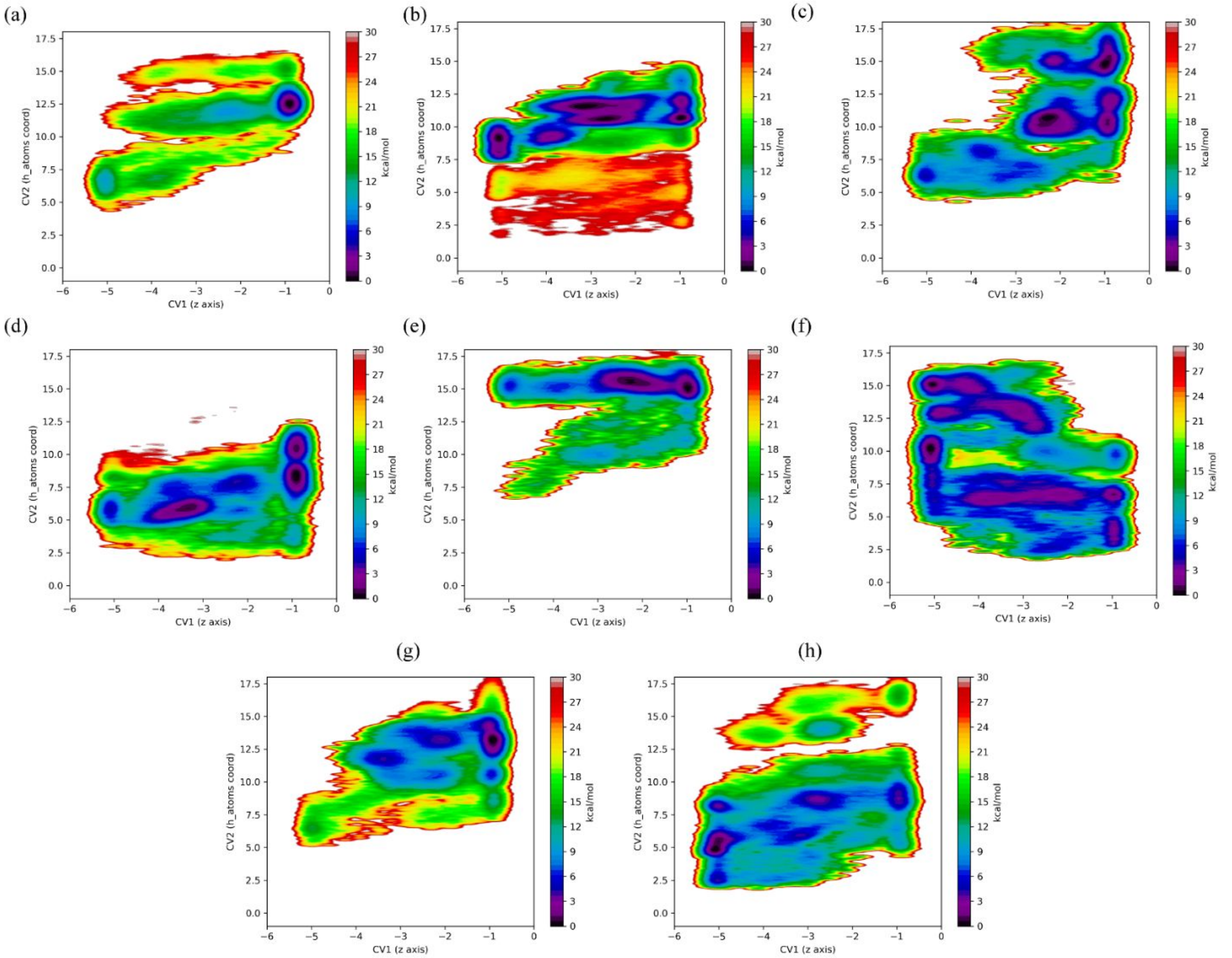

Figure S8. Free energy profiles for metal ion release, with sampling constrained to within 5 Å of the metal site toward the cytosol. CV1 represents the distance of the metal ion from the sphere's center of mass along the lipid bilayer z-axis, while CV2 indicates the metal ion coordination number (non-hydrogen atoms). Panels (a), (c), (e), and (g) show systems with standard 12-6 LJ potentials for the first to fourth setups, respectively. Panels (b), (d), (f), and (h) correspond to systems with standard 12-6-4 LJ potentials for the same setups.

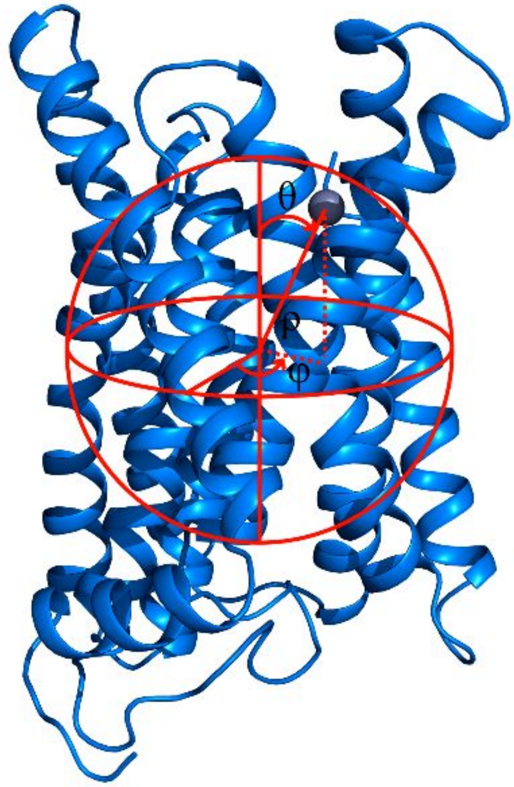

Figure S9. Sphere coordinates (ρ, θ, and ϕ) used in this study as collective variables to define the position of the metal ion during the MTD simulations.
